# Quasi-Consensus of Time-Varying Multi-Agent Systems with External Inputs under Deception Attacks

**DOI:** 10.3390/e24040447

**Published:** 2022-03-23

**Authors:** Zixian Zeng, Shiguo Peng, Wandian Feng

**Affiliations:** School of Automation, Guangdong University of Technology, Guangzhou 510006, China; 2112004108@mail2.gdut.edu.cn (Z.Z.); 2112004143@mail2.gdut.edu.cn (W.F.)

**Keywords:** quasi-consensus, time-varying multi-agent systems, external inputs, deception attacks

## Abstract

The quasi-consensus of a class of nonlinear time-varying multi-agent systems suffering from both external inputs and deception attacks is studied in this paper. This is different from a time-varying matrix, which is assumed to be bounded; further reasonable assumptions are supposed. In addition, impulsive deception attacks modeled with Bernoulli variables are considered. Sufficient conditions to achieve quasi-consensus are given, and the upper bounds of the error state related to the deception attacks is derived. Finally, a numerical simulation example is provided to show the validity of the obtained results.

## 1. Introduction

Systems with time-varying dynamics are more suitable to model objects in the real world compared with time-invariant systems. Owing to the time-varying parameters, the stability and stabilization of time-varying systems are more difficult to study even for linear systems. In recent years, results on the stability analysis of time-varying systems have been found in [1,2,3,4,5,6] and the references therein.

For example, a uniformly asymptotically stable function was proposed to study the asymptotic stability of linear time-varying systems in [5] and a nonlinear one in [6]. This approach was then extended to time-delayed systems [7], impulsive systems [8] and sampled-data systems [9]. Different from the method used in [5,6,7], the authors in [4] developed a novel lemma based on more general time-varying delayed systems.

Nevertheless, the consensus of time-varying multi-agent systems (MASs) has not been fully studied, as the analysis of time-varying systems is laborious. Moreover, time-varying dynamics in MASs mainly focus on the time delay and the communication topology [10,11,12].

For example, the uncertainty caused by unknown time-varying communication delays was considered in [10]; formation control with time-varying communication networks was studied in [12]. When studying systems with uncertain parameters, such as randomly occurring uncertainties and randomly occurring nonlinearities in [13,14,15], the norm of the time-varying term in the system matrix is always assumed to be less than one. Conservatively, this assumption is restrictive to adopt in practical works.

On the other hand, the consensus problems of MASs have been crucial issues over the past two decades [16]. The consensus of MASs means that all agents will reach the identical goal through exchanging information with neighbors [17],. It has been widely studied in practical applications, such as UAV irrigation and formation [18,19] and power grid energy distribution [20,21]. In practice, unexpected interferences, such as external inputs or cyber-attacks, may occur due to the complicated workspace. These unexpected interferences may lead to poor performance and even destroy the stability of systems. It is known that environment disturbances are inevitable and usually described by the Brownian motion.

Stochastic MASs modeled by $\mathrm{It}\hat{o}$ differential equations have been widely investigated in recent years [22]. However, the definition of consensus is not applicable if systems are disturbed by external inputs, and to describe the consensus in this case, the concept of quasi-consensus is proposed. Particularly, an error bound as controllable error for bounded external inputs is introduced in the quasi-consensus [23,24].

Significantly, working in an open environment typically brings about security problems [25]. Cyber-attacks are common, and they can destroy the stability of systems and seriously affect the equipment [26]. Hence, cyber-attacks are important factors when studying the stability of MASs. Generally, cyber-attacks are divided into two categories: denial-of-service (DoS) attacks [27,28] and deception attacks [29,30,31,32,33].

DoS attacks block communication networks, which leads to packet drops or packet time delays. For instance, the problem of communication topologies interrupted by DoS attacks with a definite probability was considered in [28].

Deception attacks relate to the situation that malicious adversaries can find and manipulate the transmitting information and control signal. False data injection attacks mean that false information can be injected to the controller or the feedback channel of the controller [29]. Furthermore, deception attacks can be divided into different categories: data replacement attacks, false data injection attacks and so on [30]. In [32], the quasi-consensus of a class of discrete-time multi-agent systems was studied using recursive linear matrix inequality and the stochastic analysis method.

In [33], in order to avoid certain adverse effects caused by system instability, load shedding and false data injection attacks, the authors designed a load frequency controller to provide a constant and uniform frequency in different operation cases of microgrids. The consensus of MASs in a given finite horizon were studied when the systems suffered from false data injection attacks [34].

To describe the randomness of deception attacks, Bernoulli variables were introduced in an attack scenario in [35]. Nevertheless, most of the existing results are on time-invariant MASs subjected to continuous-time deception attacks [29,31,32,33], while it is more practical and challenging for time-varying multi-agent systems under impulsive deception attacks.

With the above analysis, a consensus of time-varying MASs subject to both external inputs and deception attacks is missing in the literature, and how to release the restrictive assumption on the time-varying system matrix is meaningful and challenging. This paper aims to deal with the above problems, and the main contributions of this paper are summarized as follows:

(1) Compared with the traditional assumption on the time-varying system matrix of MASs, more general and practical conditions are considered in this paper versus the analysis approaches used in [5].
(2) Both false data injection attacks modeled with Bernoulli variables and external inputs are considered in this paper. Moreover, sufficient conditions for achieving the quasi-consensus are derived, and the error upper bounds related to the external inputs and deception attacks are also obtained.

The rest of this article is organized as follows. Some preliminaries are given in Section 2. Sufficient conditions for the quasi-consensus are provided in Section 3. In Section 4, an illustrative example is provided to verify the effectiveness of the proposed results. Finally, our conclusions are drawn in Section 5.

**Notation** **1.** *Throughout this article, the following notations are adopted. $\lambda_{\max}\left(\cdot \right)$ and $\lambda_{\min}\left(\cdot \right)$ are the maximum and minimum eigenvalues of any real and symmetrical matrix, respectively. $I_{n}$ and $1_{N}$ denote the n-dimensional identity matrix and an N-dimensional column vector with all ones, respectively. N={1,2,…}, N[1,N]={1,2,…,N}, where N∈N. R=(−∞,+∞), $R^{+}=\left[0,+\infty )\right.$, $R^{n}$ denotes an n-dimensional Euclidean space, and $R^{n\times m}$ is the set of n×m real matrix. diag $\left\{\cdots \right\}$ denotes a block-diagonal matrix. E[·] is the operator of expectation, and $∥x∥=\sqrt{\sum {}_{i=1}^{n}x_{i}^{2}}$ denotes the Euclidean norm of vector $x\in R^{n}$. C(X;Y) indicates the continuous mapping from X to Y. $D^{+}g\left(t\right)$ denotes the Dini derivative of the function g:R→R, and it is defined as:*

$$\begin{array}{c} D^{+}g\left(t\right)=\lim_{\Delta \to 0^{+}}\frac{g\left(t+\Delta \right)-g\left(t\right)}{\Delta }. \end{array}$$

## 2. Preliminaries

### 2.1. Graph Theory

In general, a weighted undirected graph can be represented by G=(V,E,A), where $V=\{v_{1},v_{2},\ldots ,v_{N}\}$, E⊆V×V and A = ${\left[a_{ij}\right]}_{N\times N}\in R^{N\times N}$, $v_{i},v_{j}\in V$ mean the set of vertexes, the set of edges and the adjacency matrix, respectively. When data can be transferred between agent *i* and *j*, i≠j, i,j∈N[1,N], there exists an edge between $v_{i}$ and $v_{j}$, that is $(v_{i},v_{j})\in E$, then $a_{ij}=1$ and $a_{ii}=0$, otherwise $a_{ij}=0$. *L* is Laplacian matrix of graph G, which can be denoted $L={\left[l_{ij}\right]}_{N\times N}\in R^{N\times N}$, and $l_{ij}=-a_{ij}$, i≠j, $\;l_{ii}=\sum {}_{j=1,j\neq i}^{N}a_{ij}$.

### 2.2. The Model of MASs

Consider a class of nonlinear time-varying MASs composed of *N* agents with external disturbances. The dynamics of agent i,i∈N[1,N] can be described by

(1) $$\left\{\begin{array}{c} {\dot{x}}_{i}=A\left(t\right)x_{i}\left(t\right)+\beta \left(t\right)f\left(t,x_{i}\left(t\right)),\omega_{i}\left(t\right)\right)+u_{i}\left(t\right),\;t\geq t_{0},\\ x_{i}\left(t_{0}\right)=\zeta_{i}. \end{array}\right.$$

where $x_{i}\left(t\right)\in R^{n}$, $u_{i}\left(t\right)\in R^{n}$ and $\omega_{i}\left(t\right)\in R^{n}$ represent the state, the control input and external disturbances of agent *i*, respectively. A(t)=A+α(t)TB(t)Q is a time-varying matrix, where *A*, *T*, and *Q* are constant matrices with suitable dimensions, and B(t) is a time-varying matrix. $f\left(\cdot \right)\in C\left(R^{+}\times R^{n}\times R^{n};R^{n}\right)$ is a nonlinear function, represents intrinsic dynamics of the agent. Assume that the initial time $t_{0}\geq 0$, the initial state of agent *i* is $\zeta_{i}$ and $\hat{\zeta }={\left(\zeta_{1}^{T},\zeta_{2}^{T},\ldots ,\zeta_{N}^{T}\right)}^{T}$.

The system considered in this article may be linear or non-linear, which mainly depends on the value of β(t). Clearly, while β(t)≡0, (1) is a linear system.

**Assumption** **1.** *Assume that the random variables α(t) and β(t) in system (1) both obey the Bernoulli distribution with the value 0 or 1. Their probabilities are set as follows:*

(2) $$\left\{\begin{array}{c} Pr\left(\alpha (t)=1\right)=\alpha ,\;\;Pr\left(\alpha (t)=0\right)=1-\alpha ,\\ Pr\left(\beta (t)=1\right)=\beta ,\;\;Pr\left(\beta (t)=0\right)=1-\beta . \end{array}\right.$$

*where α, β∈[0,1] are known constants. In addition, α(t) and β(t) are independent of each other.*

Based on the above conditions, the following equations are established:

(3) $$\;E\left[\alpha (t)-\alpha \right]=0,\;E\left[\beta (t)-\beta \right]=0.$$

In this article, a controller that suffers from false data injection attacks is considered, and it is designed as follows:

(4) $$\begin{array}{cc} u_{i}\left(t\right)= & \sum_{k=1}^{\infty }[U_{k}\sum_{j=1}^{N}a_{ij}\left(x_{i}\left(t\right)-x_{j}\left(t\right)\right)+\psi_{i}\left(t\right)d_{k}\xi_{i}\left(t\right)]\delta \left(t-t_{k}\right),\;\forall i\in N\left[1,N\right], \end{array}$$

where $\delta \left(\cdot \right)$ is the Dirac function, $\xi_{i}\left(t\right)$ denotes the attack signal of the agent *i*, and $d_{k}$ means the strength of attack signal at the *k*th impulsive moment. $U_{k}\in R$ is the impulsive control gain. ${\left\{t_{k}\right\}}_{k=1}^{+\infty }$ is the impulsive time sequence and satisfies $0\leq t_{0}<t_{1}<\cdots <t_{k}<\cdots$, $\lim_{k\to +\infty }t_{k}=+\infty$. Let $\tau_{\sup}=\sup_{k\in N}\left\{t_{k+1}-t_{k}\right\}$ and $\tau_{\inf}=\inf_{k\in N}\left\{t_{k+1}-t_{k}\right\}>0$. $\psi_{i}\left(t\right)$ is a Bernoulli variable related to agent *i*, which is introduced to denote the occurrence of an achievable attack. Hence, one finds:

$$\;Pr\left(\psi_{i}\left(t\right)=1\right)={\bar{\psi }}_{i},\;Pr\left(\psi_{i}\left(t\right)=0\right)=1-{\bar{\psi }}_{i}.$$

where ${\bar{\psi }}_{i}\in \left[0,1\right]$ are known constants.

**Assumption** **2.** *The stochastic variables $\psi_{i}\left(t_{k}\right)$, i∈N[1,N] are mutually independent.*

**Figure 1:**
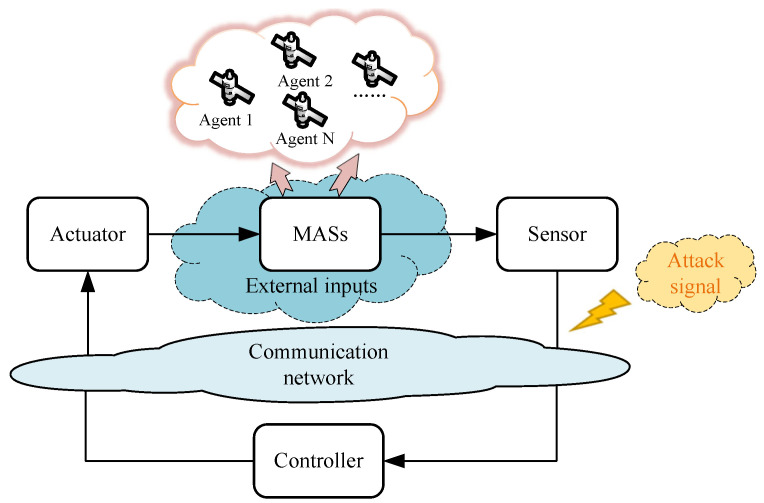
Configuration of time-varying MASs with external inputs under deception attacks.

**Remark** **1.** *The configuration of MAS with external inputs under deception attacks in this paper is shown in Figure 1. Deception attacks occur on the channel from sensor to controller, and the attackers inject false data to control signal at discrete-time instants, thus, reducing the accuracy of the system data.*

According to the controller (4), MASs (1) suffering from false data injection attacks can be described as:

(5) $$\left\{\begin{array}{c} {\dot{x}}_{i}=A\left(t\right)x_{i}\left(t\right)+\beta \left(t\right)f\left(t,x_{i}\left(t\right),\omega_{i}\left(t\right)\right),\;t\geq t_{0},\;t\neq t_{k},\\ \Delta x_{i}\left(t_{k}\right)=U_{k}\sum_{j=1}^{N}a_{ij}\left(x_{i}\left(t_{k}\right)-x_{j}\left(t_{k}\right)\right)+\psi_{i}\left(t_{k}\right)d_{k}\xi_{i}\left(t_{k}\right),\;t=t_{k}. \end{array}\right.$$

where $\Delta x_{i}\left(t_{k}\right)=x_{i}\left(t_{k}^{+}\right)-x_{i}\left(t_{k}^{-}\right)$. Throughout the article, assume that $x_{i}\left(t\right)$ is right-hand continuous at $t=t_{k}$, $x_{i}\left(t_{k}\right)=x_{i}\left(t_{k}^{+}\right)=\lim_{c\to 0^{+}}x_{i}\left(t_{k}+c\right)$ and $x_{i}\left(t_{k}^{-}\right)=\lim_{c\to 0^{-}}x_{i}\left(t_{k}+c\right)$.

Notice that the stabilization problem of an error system is equal to the consensus of MASs. Therefore, define the error state $e_{i}\left(t\right)\;:=\;x_{i}\left(t\right)-\bar{x}\left(t\right)\;=\;x_{i}\left(t\right)-\frac{1}{N}\sum_{i=1}^{N}x_{i}\left(t\right)$ and ${\bar{\omega }}_{i}\left(t\right)\;:=\;\omega_{i}\left(t\right)-\frac{1}{N}\sum_{i=1}^{N}\omega_{i}\left(t\right)$. With the help of a Kronecker product, one yields $e\left(t\right)\;=\;\left(E⊗I_{n}\right)x\left(t\right)=\left(\left(I_{N}-\frac{1}{N}1_{N}1_{N}^{T}\right)⊗I_{n}\right)x\left(t\right)$ and $\bar{\omega }\left(t\right)=\left(E⊗I_{n}\right)\omega \left(t\right)=\left(\left(I_{N}-\frac{1}{N}1_{N}1_{N}^{T}\right)⊗I_{n}\right)\omega \left(t\right)$, where $x\left(t\right)={\left[x_{1}^{T}\left(t\right),x_{2}^{T}\left(t\right),\ldots ,x_{N}^{T}\left(t\right)\right]}^{T}$, $e\left(t\right)={\left[e_{1}^{T}\left(t\right),e_{2}^{T}\left(t\right),\ldots ,e_{N}^{T}\left(t\right)\right]}^{T}$, $\omega \left(t\right)={\left[\omega_{1}^{T}\left(t\right),\omega_{2}^{T}\left(t\right),\ldots ,\omega_{N}^{T}\left(t\right)\right]}^{T}$ and $\bar{\omega }\left(t\right)={\left[{\bar{\omega }}_{1}^{T}\left(t\right),{\bar{\omega }}_{2}^{T}\left(t\right),\ldots ,{\bar{\omega }}_{N}^{T}\left(t\right)\right]}^{T}$.

Then, the compact form error system under the false data injection attacks can be described as:

(6) $$\left\{\begin{array}{c} \dot{e}\left(t\right)=\left(I_{N}⊗A\left(t\right)\right)e\left(t\right)+\beta \left(t\right)F\left(t,e\left(t\right),\bar{\omega }\left(t\right)\right),\;t\geq t_{0},\;t\neq t_{k},\\ \Delta e\left(t_{k}\right)=e\left(t_{k}^{+}\right)-e\left(t_{k}^{-}\right)=\left(U_{k}L⊗I_{n}\right)e\left(t_{k}^{-}\right)+d_{k}\left(E\Psi \left(t_{k}\right)⊗I_{n}\right)\xi \left(t_{k}\right),\;\;t=t_{k}. \end{array}\right.$$

where $\Psi \left(t_{k}\right)=\mathrm{diag}\left\{\psi_{1}\left(t_{k}\right),\psi_{2}\left(t_{k}\right),\ldots ,\psi_{N}\left(t_{k}\right)\right\}$ is a diagonal matrix, $\xi \left(t_{k}\right)={\left[\xi_{1}^{T}\left(t_{k}\right),\xi_{2}^{T}\left(t_{k}\right),\ldots ,\xi_{N}^{T}\left(t_{k}\right)\right]}^{T}$ as well as $F\left(t,e\left(t\right),\bar{\omega }\left(t\right)\right)=\left(E⊗I_{n}\right)f\left(t,x\left(t\right),\omega \left(t\right)\right)$, where $f\left(t,x\left(t\right),\omega \left(t\right)\right)={\left[f^{T}\left(t,x_{1}\left(t\right),\omega_{1}\left(t\right)\right),\ldots ,f^{T}\left(t,x_{N}\left(t\right),\omega_{N}\left(t\right)\right)\right]}^{T}$.

**Assumption** **3.** *The attack signal $\xi (t_{k})$, k∈N, is bound: ${∥\xi (t_{k})∥}^{2}<\bar{\xi }$, and $\bar{\xi }$ is a known positive constant.*

**Assumption** **4.** *For the nonlinear function f in system* (1), *there exist constants $\kappa_{1}$, $\kappa_{2}\geq 0$ that satisfy*

$$\begin{array}{cc} |f^{T}\left(t,x_{1}\left(t\right),y_{1}\left(t\right)\right)- & f^{T}\left(t,x_{2}\left(t\right),y_{2}\left(t\right)\right)|\;\times |f\left(t,x_{1}\left(t\right),y_{1}\left(t\right)\right)-f\left(t,x_{2}\left(t\right),y_{2}\left(t\right)\right)|\\ \; & \leq \kappa_{1}{\left|x_{1}\left(t\right)-x_{2}\left(t\right)\right|}^{2}+\kappa_{2}{\left|y_{1}\left(t\right)-y_{2}\left(t\right)\right|}^{2}. \end{array}$$

**Assumption** **5.** *Assume that the external disturbance $\bar{\omega }\left(t\right)$ is bounded and there exists a positive constant $\hat{\omega }$ that satisfies*

$$\begin{array}{c} \underset{t\geq t_{0}}{\sup}∥\bar{\omega }\left(t\right)∥\leq \hat{\omega }, \end{array}$$

**Lemma** **1.** *Ref. [36] Assume that ι∈R,Γ,Υ,ZandΛ are matrices with proper dimensions. Therefore, the Kronecker product has the properties*

$$\begin{array}{c} \left(1\right)\;\;{\left(\Gamma ⊗\Upsilon \right)}^{T}=\Gamma^{T}⊗\Upsilon^{T};\\ (2)\;\;(\iota \Gamma )⊗\Upsilon =\Gamma ⊗(\iota \Upsilon );\\ (3)\;\;(\Gamma +Z)⊗\Upsilon =\Gamma ⊗\Upsilon +Z⊗\Upsilon ;\\ (4)\;\;(\Gamma ⊗Z)(\Upsilon ⊗\Lambda )=(\Gamma \Upsilon )⊗Z\Lambda . \end{array}$$

**Definition** **1.** *Ref. [37] For any given initial value of the system $\hat{\zeta }$, if there exists a compact set $\hat{\partial }$ and a constant ϑ such that as t→+∞, the error state e(t) converges to*

(7) $$\hat{\partial }=\left\{e\in R^{nN}|E\left[∥e∥\right]\leq \vartheta \right\},$$

*and then the MASs (1) is said to achieve quasi-consensus; ϑ is the upper bound of error; and if ϑ=0, MASs (1) is said to achieve consensus.*

**Definition** **2.** *Ref. [8] Given an impulsive sequence $\tau^{\prime }={\left\{t_{k}\right\}}_{k=1}^{+\infty },k\in N$, let $N_{\tau^{\prime }}\left(t,s\right)$ denotes the number of impulsive times in the interval (s,t], exists two constants $N_{0}\in N$ and $\tau_{ave}>0$, such that*

(8) $$N_{\tau^{\prime }}\left(t,s\right)\leq \frac{t-s}{\tau_{ave}}+N_{0}.$$

*$\tau_{ave}$ and $N_{0}$ are called the average impulsive interval and the elasticity number, respectively.*

## 3. Main Results

**Theorem** **1.** *Suppose that Assumptions 1–5 hold, if there exists a positive definite matrix P and positive scalars $\varepsilon_{1}$, $\varepsilon_{2}$, $\varepsilon_{3}$, γ, $\kappa_{1}$, $\kappa_{2}$ and $\gamma_{0}\geq 0$, such that*

(9) $$\int_{s}^{t}H(v)dv\leq \gamma \left(t-s\right)+\gamma_{0},\;0\leq s<t,$$

(10) $$\theta =\frac{\ln(Z_{\sup}\sigma )}{\tau_{\sup}}+\gamma <0,$$

(11) 0<σ<1,

*where $\bar{\Lambda }\left(t\right)=P\left(I_{N}⊗A\right)+{\left(I_{N}⊗A\right)}^{T}P+\alpha \left[P\left(I_{N}⊗TB\left(t\right)Q\right)+{\left(I_{N}⊗TB\left(t\right)Q\right)}^{T}P\right]+\beta \varepsilon_{1}PP+\beta \kappa^{2}\varepsilon_{1}^{-1}\left(\varepsilon_{2}+1\right)I_{nN}$, $b=\lambda_{\min}\left(P\right)$, $q=\lambda_{\max}\left(P\right)$, $H\left(t\right)=b^{-1}\lambda_{\max}\left(\bar{\Lambda }\left(t\right)\right)$, $\eta ={\hat{d}}^{2}\bar{\xi }\bar{\psi }q\left(\varepsilon_{3}^{-1}+1\right)$, $\sigma =d^{2}\left(\varepsilon_{3}qb^{-1}+1\right)$, $\rho =\beta \kappa^{2}\varepsilon_{1}^{-1}\left(\varepsilon_{2}^{-1}+1\right)$, $\bar{\psi }=\max_{i\in N[1,N]}{\bar{\psi }}_{i}$, $\hat{d}=\max_{k\in N}d_{k}$, $G=\left(I_{N}+U_{k}L\right)⊗I_{n}$, $d=\lambda_{\max}\left(G\right)$, $Z_{\sup}=e^{\gamma \tau_{\sup}+\gamma_{0}}$ and $\kappa =\max\{\kappa_{1},\kappa_{2}\}$.*

*Then, the time-varying multi-agent system (1) with external inputs and deception attacks can achieve quasi-consensus under the control protocol (4), and the upper bound of error can be estimated as*

$$\vartheta =\frac{\rho \sigma^{N_{0}}e^{\gamma_{0}}{\hat{\omega }}^{2}}{b\left|\theta \right|}+\frac{\eta Z_{\sup}}{b(1-\sigma Z_{\sup})}+b^{-1}\rho Z_{\sup}{\hat{\omega }}^{2}\tau_{\sup},$$

*where $Z_{\sup}=e^{\gamma \tau_{\sup}+\gamma_{0}}$.*

**Proof.**  Consider the following Lyapunov function:

(12) $$V\left(t,e\left(t\right)\right)=e^{T}\left(t\right)Pe\left(t\right).$$

For $t\in [t_{k},t_{k+1})$, k∈N, taking the Dini derivative of (12) gives:

(13) $$\begin{array}{c} D^{+}V\left(t,e\left(t\right)\right)=2e^{T}\left(t\right)P\left[\left(I_{N}⊗A\left(t\right)\right)e\left(t\right)+\beta \left(t\right)F\left(t,e\left(t\right),\bar{\omega }\left(t\right)\right)\right]. \end{array}$$

According to Assumption 1, one has:

(14) $$\begin{array}{cc} 2e^{T}\left(t\right)P\left(I_{N}⊗A\left(t\right)\right)e\left(t\right)= & \;e^{T}\left(t\right)\left(P\left(I_{N}⊗A\right)+{\left(I_{N}⊗A\right)}^{T}P\right)e\left(t\right)+\alpha e^{T}\left(t\right)(P\left(I_{N}⊗TB\left(t\right)Q\right)\\ \; & +{\left(I_{N}⊗TB\left(t\right)Q\right)}^{T}P)e\left(t\right)+\left(\alpha \left(t\right)-\alpha \right)e^{T}\left(t\right)(P\left(I_{N}⊗TB\left(t\right)Q\right)\\ \; & +{\left(I_{N}⊗TB\left(t\right)Q\right)}^{T}P)e\left(t\right). \end{array}$$

(15) $$\begin{array}{cc} 2e^{T}\left(t\right)P\beta \left(t\right)F\left(t,e\left(t\right),\bar{\omega }\left(t\right)\right)= & \;\beta [e^{T}\left(t\right)PF\left(t,e\left(t\right),\bar{\omega }\left(t\right)\right)+F^{T}\left(t,e\left(t\right),\bar{\omega }\left(t\right)\right)Pe\left(t\right)]\\ \; & +2\left(\beta \left(t\right)-\beta \right)e^{T}\left(t\right)PF\left(t,e\left(t\right),\bar{\omega }\left(t\right)\right). \end{array}$$

Based on Assumption 4, it can be found that:

(16) $$\begin{array}{cc} F^{T}\left(t,e\left(t\right),\bar{\omega }\left(t\right)\right) & F\left(t,e\left(t\right),\bar{\omega }\left(t\right)\right)\;=f^{T}\left(t,x\left(t\right),\omega \left(t\right)\right)(E⊗I_{n}{)}^{T}\left(E⊗I_{n}\right)f\left(t,x\left(t\right),\omega \left(t\right)\right)\\ & \;\leq \left[\kappa_{1}x^{T}\left(t\right)+\kappa_{2}\omega^{T}\left(t\right)\right]\left(E⊗I_{n}\right)\left(E⊗I_{n}\right)\left[\kappa_{1}x\left(t\right)+\kappa_{2}\omega \left(t\right)\right]\\ \; & \;=\kappa_{1}^{2}e^{T}\left(t\right)e\left(t\right)+\kappa_{1}\kappa_{2}\left[{\bar{\omega }}^{T}\left(t\right)e\left(t\right)+e^{T}\left(t\right)\bar{\omega }\left(t\right)\right]+\kappa_{2}^{2}{\bar{\omega }}^{T}\left(t\right)\bar{\omega }\left(t\right)\\ \; & \;\leq \left(\kappa_{1}^{2}+\kappa_{1}\kappa_{2}\varepsilon_{2}\right)e^{T}\left(t\right)e\left(t\right)+\left(\kappa_{2}^{2}+\kappa_{1}\kappa_{2}\varepsilon_{2}^{-1}\right){\bar{\omega }}^{T}\left(t\right)\bar{\omega }\left(t\right), \end{array}$$

(17) $$\begin{array}{cc} \beta [e^{T}\left(t\right)PF\left(t,e\left(t\right),\bar{\omega }\left(t\right)\right) & +F^{T}\left(t,e\left(t\right),\bar{\omega }\left(t\right)\right)Pe\left(t\right)].\\ \; & \;\leq \beta [\varepsilon_{1}e^{T}\left(t\right)PPe\left(t\right)+\varepsilon_{1}^{-1}F^{T}\left(t,e\left(t\right),\bar{\omega }\left(t\right)\right)F\left(t,e\left(t\right),\bar{\omega }\left(t\right)\right)]\\ \; & \;\leq \beta [\varepsilon_{1}e^{T}\left(t\right)PPe\left(t\right)+\left(\kappa_{1}^{2}\varepsilon_{1}^{-1}+\kappa_{1}\kappa_{2}\varepsilon_{2}\varepsilon_{1}^{-1}\right)e^{T}\left(t\right)e\left(t\right)\\ \; & \;+\left(\kappa_{2}^{2}\varepsilon_{1}^{-1}+\kappa_{1}\kappa_{2}\varepsilon_{2}^{-1}\varepsilon_{1}^{-1}\right){\bar{\omega }}^{T}\left(t\right)\bar{\omega }\left(t\right)]. \end{array}$$

Substituting (14)–(17) into (13) and taking the mathematical expectation operation gives:

(18) $$\begin{array}{cc} E[D^{+} & V\left(t,e\left(t\right)\right)]\leq E\{e^{T}\left(t\right)\left(P\left(I_{N}⊗A\right)+{\left(I_{N}⊗A\right)}^{T}P\right)e\left(t\right)+\alpha e^{T}\left(t\right)[P\left(I_{N}⊗TB\left(t\right)Q\right)\\ \; & +{\left(I_{N}⊗TB\left(t\right)Q\right)}^{T}P]e\left(t\right)+\beta \varepsilon_{1}e^{T}\left(t\right)PPe\left(t\right)+\left(\beta \kappa_{1}^{2}\varepsilon_{1}^{-1}+\beta \kappa_{1}\kappa_{2}\varepsilon_{2}\varepsilon_{1}^{-1}\right)e^{T}\left(t\right)e\left(t\right)\\ \; & +\left(\beta \kappa_{2}^{2}\varepsilon_{1}^{-1}+\beta \kappa_{1}\kappa_{2}\varepsilon_{2}^{-1}\varepsilon_{1}^{-1}\right){\bar{\omega }}^{T}\left(t\right)\bar{\omega }\left(t\right)\}. \end{array}$$

Therefore, combining the above conditions with (18), we have:

(19) $$\begin{array}{c} E\left[D^{+}V\left(t,e\left(t\right)\right)\right]\leq H\left(t\right)E\left[V\left(t,e\left(t\right)\right)\right]+\rho {\bar{\omega }}^{T}\left(t\right)\bar{\omega }\left(t\right), \end{array}$$

where $\bar{\Lambda }\left(t\right)=P\left(I_{N}⊗A\right)+{\left(I_{N}⊗A\right)}^{T}P+\alpha \left[P\left(I_{N}⊗TB\left(t\right)Q\right)+{\left(I_{N}⊗TB\left(t\right)Q\right)}^{T}P\right]+\beta \varepsilon_{1}PP$ + $\beta \kappa^{2}\varepsilon_{1}^{-1}\left(\varepsilon_{2}+1\right)I_{nN}$, $b=\lambda_{\min}\left(P\right)$, $H\left(t\right)=b^{-1}\lambda_{\max}\left(\bar{\Lambda }\left(t\right)\right)$, $\rho =\beta \kappa^{2}\varepsilon_{1}^{-1}\left(\varepsilon_{2}^{-1}+1\right)$ and $\kappa =\max\{\kappa_{1},\kappa_{2}\}$.

For $t\in [t_{k},t_{k+1})$, k∈N and any positive number *S*, we establish a comparative differential equation as follows:

(20) $$\left\{\begin{array}{c} \dot{y}\left(t\right)=H\left(t\right)y\left(t\right)+\rho {\bar{\omega }}^{T}\left(t\right)\bar{\omega }\left(t\right)+S,\\ y\left(t_{k}\right)=\Phi_{k} \end{array}\right.$$

where $\Phi_{k}=E\left[V\left(t_{k},e\left(t_{k}\right)\right)\right]+S$ By solving and comparing the solutions of the differential equations, the following result is obtained:

$$\begin{array}{cc} E[V_{S}\left(t,e\left(t\right)\right)]\leq \; & \Phi_{k}e^{\int_{t_{k}}^{t}H(v)dv}+\rho \int_{t_{k}}^{t}\left({\bar{\omega }}^{T}\left(u\right)\bar{\omega }\left(u\right)+S\right)e^{\int_{u}^{t}H(v)dv}du. \end{array}$$

For $t\in [t_{k},t_{k+1})$, k∈N, setting S→0, one has:

(21) $$\begin{array}{cc} E[V(t,e(t))]\leq & \;E\left[V\left(t_{k},e\left(t_{k}\right)\right)\right]e^{\int_{t_{k}}^{t}H(v)dv}+\rho \int_{t_{k}}^{t}{\bar{\omega }}^{T}\left(u\right)\bar{\omega }\left(u\right)e^{\int_{u}^{t}H(v)dv}du. \end{array}$$

For $t=t_{k}$, k∈N, according to (6), we obtain:

(22) $$\begin{array}{cc} E[V\left(t_{k}^{+},e\left(t_{k}^{+}\right)\right)] & =E\left[e^{T}\left(t_{k}^{+}\right)Pe\left(t_{k}^{+}\right)\right]=\;E\left[\left(e^{T}\left(t_{k}^{-}\right)G^{T}+W^{T}\left(t_{k}\right)\right)\times P\left(Ge\left(t_{k}^{-}\right)+W\left(t_{k}\right)\right)\right]\\ \; & =E[e^{T}\left(t_{k}^{-}\right)G^{T}PGe\left(t_{k}^{-}\right)+e^{T}\left(t_{k}^{-}\right)G^{T}PW\left(t_{k}\right)+W^{T}\left(t_{k}\right)PGe\left(t_{k}^{-}\right)\\ \; & \;+W^{T}\left(t_{k}\right)PW\left(t_{k}\right)], \end{array}$$

where $W\left(t_{k}\right)=d_{k}\left(E\Psi \left(t_{k}\right)⊗I_{n}\right)\xi \left(t_{k}\right)$. As for the first term in (22), has:

(23) $$\begin{array}{cc} E[e^{T}\left(t_{k}^{-}\right)G^{T}PGe\left(t_{k}^{-}\right)]=\; & E\{e^{T}\left(t_{k}^{-}\right)\left[\left(I_{N}+U_{k}L^{T}\right)⊗I_{n}\right]P\left[\left(I_{N}+U_{k}L\right)⊗I_{n}\right]e\left(t_{k}^{-}\right)\}\\ \leq \; & d^{2}E\left[V\left(t_{k}^{-},e\left(t_{k}^{-}\right)\right)\right]. \end{array}$$

Then, we have:

(24) $$\begin{array}{cc} E[e^{T}\left(t_{k}^{-}\right)G^{T}PW\left(t_{k}\right)+ & \;W^{T}\left(t_{k}\right)PGe\left(t_{k}^{-}\right)]\leq qE\left[e^{T}\left(t_{k}^{-}\right)G^{T}W\left(t_{k}\right)+\;W^{T}\left(t_{k}\right)Ge\left(t_{k}^{-}\right)\right]\\ \leq \; & qE[\varepsilon_{3}e^{T}\left(t_{k}^{-}\right)G^{T}Ge\left(t_{k}^{-}\right)+\varepsilon_{3}^{-1}W^{T}\left(t_{k}\right)W\left(t_{k}\right)]\\ \leq & \varepsilon_{3}d^{2}qb^{-1}E\left[V\left(t_{k}^{-},e\left(t_{k}^{-}\right)\right)\right]+\varepsilon_{3}^{-1}{\hat{d}}^{2}\bar{\xi }\bar{\psi }q. \end{array}$$

For the fourth term, we obtain:

(25) $$\begin{array}{cc} E[W^{T}\left(t_{k}\right)PW\left(t_{k}\right)]= & \;E\left[d_{k}^{2}\xi^{T}\left(t_{k}\right){\left(E\Psi \left(t_{k}\right)⊗I_{n}\right)}^{T}P\left(E\Psi \left(t_{k}\right)⊗I_{n}\right)\xi \left(t_{k}\right)\right]\leq {\hat{d}}^{2}\bar{\xi }\bar{\psi }q. \end{array}$$

In summary, through (23)–(25), we reach the following conclusion:

(26) $$\begin{array}{cc} E[V\left(t_{k}^{+},e\left(t_{k}^{+}\right)\right)]\leq & \;d^{2}\left(\varepsilon_{3}qb^{-1}+1\right)E[V\left(t_{k}^{-},e\left(t_{k}^{-}\right)\right)+{\hat{d}}^{2}\bar{\xi }\bar{\psi }q\left(\varepsilon_{3}^{-1}+1\right)\\ \leq & \;\sigma E[V\left(t_{k}^{-},e\left(t_{k}^{-}\right)\right)]+\eta , \end{array}$$

where $\sigma =d^{2}\left(\varepsilon_{3}qb^{-1}+1\right)$ and $\eta ={\hat{d}}^{2}\bar{\xi }\bar{\psi }q\left(\varepsilon_{3}^{-1}+1\right)$.

In this part, the mathematical induction method will be used to obtain the overall evolution process of the system, based on (21), which is proven as follows:

For $t\in [t_{0},t_{1})$, we have

(27) $$\begin{array}{c} E\left[V\left(t,e\left(t\right)\right)\right]\leq E\left[V\left(t_{0},e\left(t_{0}\right)\right)\right]e^{\int_{t_{0}}^{t}H(v)dv}+\rho \int_{t_{0}}^{t}{\bar{\omega }}^{T}\left(u\right)\bar{\omega }\left(u\right)e^{\int_{u}^{t}H(v)dv}du. \end{array}$$

According to (21) and (26), for any $t\in [t_{m-1},t_{m})$, m∈N, suppose that the following inequality holds:

(28) $$\begin{array}{cc} E[V(t,e(t))] & \leq \sigma^{m-1}E\left[V\left(t_{0},e\left(t_{0}\right)\right)\right]e^{\int_{t_{0}}^{t}H(v)dv}+\sum_{i=0}^{m-2}(\sigma^{m-i-1}\rho \int_{t_{i}}^{t_{i+1}}{\bar{\omega }}^{T}\left(u\right)\bar{\omega }\left(u\right)\\ \; & \times e^{\int_{u}^{t}H(v)dv}du+\sigma^{i}\eta e^{\int_{t_{m-i-1}}^{t}H(v)dv})+\rho \int_{t_{m-1}}^{t}{\bar{\omega }}^{T}\left(u\right)\bar{\omega }\left(u\right)e^{\int_{u}^{t}H(v)dv}du. \end{array}$$

For $t\in [t_{m},t_{m+1})$, m∈N, a comparative differential equation similar to (20) is established as follows:

(29) $$\left\{\begin{array}{c} \dot{y}\left(t\right)=H\left(t\right)y\left(t\right)+\rho {\bar{\omega }}^{T}\left(t\right)\bar{\omega }\left(t\right)+S,\\ y\left(t_{m}\right)=\Phi_{m}. \end{array}\right.$$

where $\Phi_{m}=\sigma E\left[V\left(t_{m},e\left(t_{m}\right)\right)\right]+S$. By solving and comparing the solutions of the differential equations and S→0, the following result is obtained:

(30) $$\begin{array}{cc} E[V(t,e(t))] & \leq \sigma E\left[V\left(t_{m},e\left(t_{m}\right)\right)\right]e^{\int_{t_{m}}^{t}H(v)dv}+\rho \int_{t_{m}}^{t}{\bar{\omega }}^{T}\left(u\right)\bar{\omega }\left(u\right)e^{\int_{u}^{t}H(v)dv}du\\ \; & \leq \sigma^{m}E\left[V\left(t_{0},e\left(t_{0}\right)\right)\right]e^{\int_{t_{0}}^{t}H(v)dv}+\sum_{i=0}^{m-1}(\sigma^{m-i}\rho \int_{t_{i}}^{t_{i+1}}{\bar{\omega }}^{T}\left(u\right)\bar{\omega }\left(u\right)\\ \; & \times \;e^{\int_{u}^{t}H(v)dv}du+\sigma^{i}\eta e^{\int_{t_{m-i}}^{t}H(v)dv})+\rho \int_{t_{m}}^{t}{\bar{\omega }}^{T}\left(u\right)\bar{\omega }\left(u\right)e^{\int_{u}^{t}H(v)dv}du. \end{array}$$

Based on the definition of $N_{\tau^{\prime }}\left(t,s\right)$ in Definition 2, Assumption 5 and (30), for any $t\geq t_{0}$, one finds:

(31) $$\begin{array}{cc} E[V(t,e(t))]\leq & \;\sigma^{N_{\tau^{\prime }}\left(t,t_{0}\right)}E\left[V\left(t_{0},e\left(t_{0}\right)\right)\right]e^{\int_{t_{0}}^{t}H(v)dv}+\rho \int_{t_{0}}^{t}\sigma^{N_{\tau^{\prime }}\left(t,u\right)}{\bar{\omega }}^{T}\left(u\right)\bar{\omega }\left(u\right)e^{\int_{u}^{t}H(v)dv}du\\ \; & +\eta Z_{\sup}\frac{1-{\left(\sigma Z_{\inf}\right)}^{N_{\tau^{\prime }}\left(t,t_{0}\right)}}{1-\sigma Z_{\sup}}+\rho Z_{\sup}{\hat{\omega }}^{2}\tau_{\sup}, \end{array}$$

where $Z_{\sup}=e^{\gamma \tau_{\sup}+\gamma_{0}}$ and $Z_{\inf}=e^{\gamma \tau_{\inf}+\gamma_{0}}$. Then, set $\theta =\frac{\ln(Z_{\sup}\sigma )}{\tau_{ave}}+\gamma <0$, and one has:

(32) $$\begin{array}{cc} E[V(t,e(t))]\leq & \sigma^{\frac{t-t_{0}}{\tau_{ave}}+N_{0}}e^{\gamma \left(t-t_{0}\right)+\gamma_{0}}E\left[V\left(t_{0},e\left(t_{0}\right)\right)\right]+\rho \int_{t_{0}}^{t}\sigma^{\frac{t-u}{\tau_{ave}}+N_{0}}e^{\gamma \left(t-u\right)+\gamma_{0}}{\bar{\omega }}^{T}\left(u\right)\bar{\omega }\left(u\right)du\\ \; & +\eta Z_{\sup}\frac{1-{\left(\sigma Z_{\inf}\right)}^{\frac{t-t_{0}}{\tau_{ave}}+N_{0}}}{1-\sigma Z_{\sup}}+\rho Z_{\sup}{\hat{\omega }}^{2}\tau_{\sup}\\ \leq & \;\sigma^{N_{0}}e^{\gamma_{0}}e^{\theta (t-t_{0})}E\left[V\left(t_{0},e\left(t_{0}\right)\right)\right]+\rho \sigma^{N_{0}}e^{\gamma_{0}}\int_{t_{0}}^{t}e^{\theta (t-u)}{\bar{\omega }}^{T}\left(u\right)\bar{\omega }\left(u\right)du\\ \; & +\eta Z_{\sup}\frac{1-{\left(\sigma Z_{\inf}\right)}^{\frac{t-t_{0}}{\tau_{ave}}+N_{0}}}{1-\sigma Z_{\sup}}+\rho Z_{\sup}{\hat{\omega }}^{2}\tau_{\sup}. \end{array}$$

It follows from (32) that:

(33) $$\begin{array}{c} \lim_{t\to +\infty }E\left[V\left(t,e\left(t\right)\right)\right]\leq \frac{\rho \sigma^{N_{0}}e^{\gamma_{0}}{\hat{\omega }}^{2}}{\left|\theta \right|}+\frac{\eta Z_{\sup}}{1-\sigma Z_{\sup}}+\rho Z_{\sup}{\hat{\omega }}^{2}\tau_{\sup}. \end{array}$$

As

$$\begin{array}{c} E\left[{∥e(t)∥}^{2}\right]⩽\frac{1}{b}E\left[V\left(t,e\left(t\right)\right)\right]. \end{array}$$

In conclusion, the nonlinear time-varying multi-agent systems with external inputs under deception attacks can achieve quasi-consensus under impulsive protocol (4), and they have the upper bound of error:

$$\vartheta =\frac{\rho \sigma^{N_{0}}e^{\gamma_{0}}{\hat{\omega }}^{2}}{b\left|\theta \right|}+\frac{\eta Z_{\sup}}{b(1-\sigma Z_{\sup})}+b^{-1}\rho Z_{\sup}{\hat{\omega }}^{2}\tau_{\sup}.$$

□

**Remark** **2.** *Different from the works [31,32,35], the deception attacks in this paper mainly focus on the false data injection attacks in impulsive form. Note that the time-varying matrix in [37] assumes that $B^{T}\left(t\right)B\left(t\right)\leq KI_{n}$ with 0<K<+∞, and this bounded condition is removed here. In [35], the quasi-consensus problem of time-invariant systems under deception attacks is considered. Compare with [35], this paper takes into account external inputs, deception attacks and time-varying dynamics, which have greater significance in practice.*

**Remark** **3.** *Based on the continuous time evolution characteristics of the system, a reasonable assumption is constructed as (9). In addition, it can be seen from (21) that the system is always unstable without a control protocol. If the system is stable, then (9) can be changed to:*

(34) $$\int_{s}^{t}H(v)dv\leq -\gamma \left(t-s\right)-\gamma_{0},\;0\leq s<t.$$

**Corollary** **1.** *Under Assumptions 1–5, if there exists a positive definite matrix P and positive scalars $\varepsilon_{1}$, $\varepsilon_{2}$, $\varepsilon_{3}$, γ, $\kappa_{1}$, $\kappa_{2}$ and $\gamma_{0}\geq 0$, the condition (34), and the following condition is satisfied:*

(35) $$\tilde{\theta }=\frac{\ln(Z_{nd}\sigma )}{\tau_{ave}}-\gamma <0,$$

*where $Z_{nd}=e^{-\gamma \tau_{\inf}-\gamma_{0}}$. Then, the nonlinear time-varying multi-agent system (1) with external inputs and deception attacks can achieve quasi-consensus under the control protocol (4), and the upper bound of error can be estimated as:*

(36) $$\vartheta^{{}^{\prime }}=\frac{\rho \sigma^{N_{0}}e^{-\gamma_{0}}{\hat{\omega }}^{2}}{b\left|\tilde{\theta }\right|}+\frac{\eta Z_{nd}}{b(1-\sigma Z_{nd})}+b^{-1}\rho Z_{nd}{\hat{\omega }}^{2}\tau_{\sup}.$$

**Proof.**  As this inference only involves the assumption of continuous time evolution characteristics of the system, it only needs to prove the first part and the third part according to Theorem 1. According to the solution of (19) and comparison function (20), for $t\in [t_{k},t_{k+1})$, k∈N, setting S→0, one can find:

(37) $$\begin{array}{cc} E[V(t,e(t))]\leq & \;E\left[V\left(t_{k},e\left(t_{k}\right)\right)\right]e^{\int_{t_{k}}^{t}H(v)dv}+\rho \int_{t_{k}}^{t}{\bar{\omega }}^{T}\left(u\right)\bar{\omega }\left(u\right)e^{\int_{u}^{t}H(v)dv}du. \end{array}$$

Similarly, we find that, as S→0

(38) $$\begin{array}{cc} E[V(t,e(t))]\leq & \;\sigma^{N_{0}}e^{-\gamma_{0}}e^{\tilde{\theta }\left(t-t_{0}\right)}E\left[V\left(t_{0},e\left(t_{0}\right)\right)\right]+\rho \sigma^{N_{0}}e^{-\gamma_{0}}\int_{t_{0}}^{t}e^{\tilde{\theta }\left(t-u\right)}{\bar{\omega }}^{T}\left(u\right)\bar{\omega }\left(u\right)du\\ \; & +\eta Z_{nd}\frac{1-{\left(\sigma Z_{\inf}^{{}^{\prime }}\right)}^{\frac{t-t_{0}}{\tau_{ave}}+N_{0}}}{1-\sigma Z_{nd}}+\rho Z_{nd}{\hat{\omega }}^{2}\tau_{\sup}, \end{array}$$

where $Z_{\inf}^{{}^{\prime }}=e^{-\gamma \tau_{\sup}-\gamma_{0}}$. When t→+∞, we have:

(39) $$\begin{array}{cc} \lim_{t\to +\infty }E[V\left(t,e\left(t\right)\right) & ]\leq \frac{\rho \sigma^{N_{0}}e^{-\gamma_{0}}{\hat{\omega }}^{2}}{\left|\tilde{\theta }\right|}+\frac{\eta Z_{nd}}{1-\sigma Z_{nd}}+\rho Z_{nd}{\hat{\omega }}^{2}\tau_{\sup}. \end{array}$$

Hence, the nonlinear time-varying multi-agent system with external inputs and deception attacks can achieve quasi-consensus under control protocol (4), and

$$\vartheta^{{}^{\prime }}=\frac{\rho \sigma^{N_{0}}e^{-\gamma_{0}}{\hat{\omega }}^{2}}{b\left|\tilde{\theta }\right|}+\frac{\eta Z_{nd}}{b(1-\sigma Z_{nd})}+b^{-1}\rho Z_{nd}{\hat{\omega }}^{2}\tau_{\sup}.$$

□

## 4. Numerical Examples

**Figure 2:**
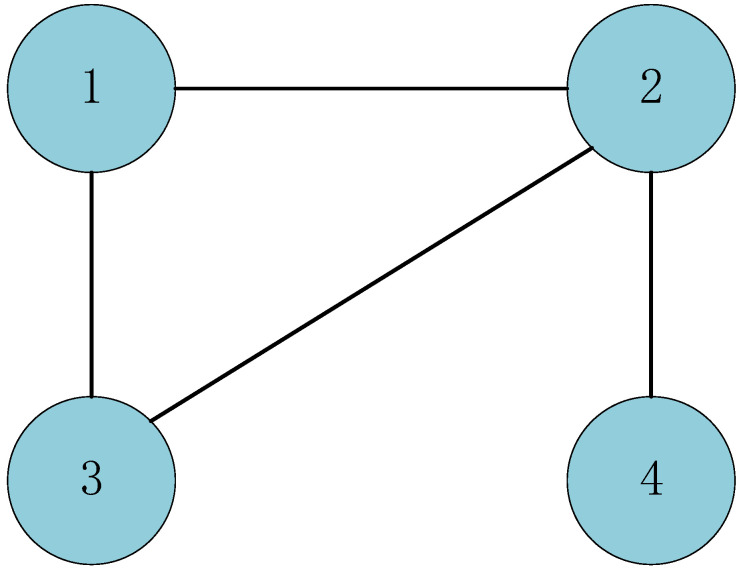
Undirected communication graph of time-varying MASs (1).

In this section, a numerical example is provided to verify the applicability of theoretical results. The undirected communication graph of time-varying MASs (1) is shown as Figure 2. From this, we know that

$$L=\left[\begin{array}{cccc} 2 & -1 & -1 & 0\\ -1 & 3 & -1 & -1\\ -1 & -1 & 2 & 0\\ 0 & -1 & 0 & 1 \end{array}\right].$$

We consider 3-dimensional time-varying MASs with four agents, i.e., $x_{i}\left(t\right)=[x_{i1}\left(t\right),$$x_{i2}\left(t\right),x_{i3}{\left(t\right)]}^{T}$ and i∈N[1,4]. Setting $\omega_{i}\left(t\right)={\left[0.15\cos\left(t\right),-0.2\sin\left(t\right),0.3\cos\left(t\right)\right]}^{T}$, $t_{0}=0$ and

$$\begin{array}{c} \hat{\zeta }={\left(\zeta_{1}^{T},\zeta_{2}^{T},\ldots ,\zeta_{4}^{T}\right)}^{T}={\left[\begin{array}{cccc} 0.7 & -0.2 & 1.3 & -0.3\\ 0.2 & -2.4 & 2.5 & 0.7\\ 1.6 & 0.4 & -0.4 & 1.4 \end{array}\right]}^{T}. \end{array}$$

**Figure 3:**
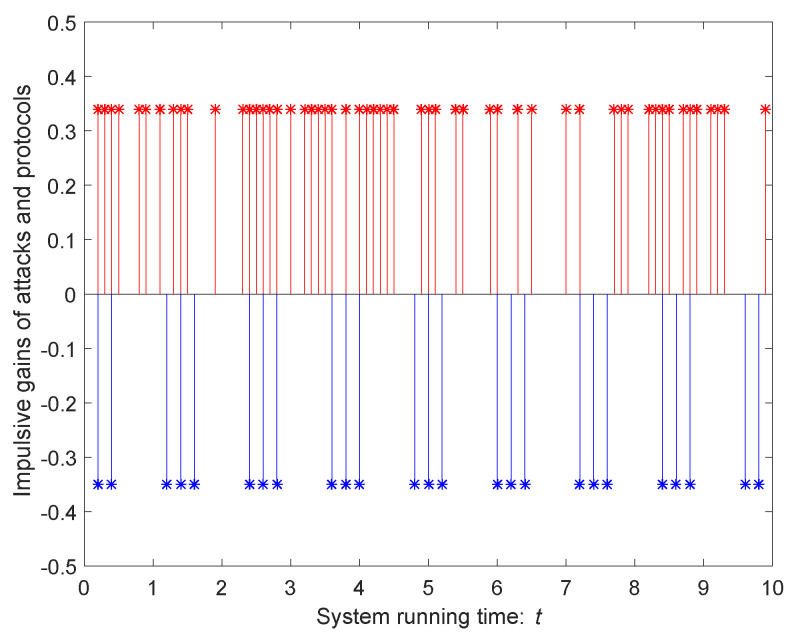
Impulsive sequence for $\hat{d}=0.34$ and $U_{k}=-0.35$.

In addition, let $f\left(t,x_{i}\left(t\right),\omega_{i}\left(t\right)\right)={\left[\mathrm{sat}\left(x_{i1}\left(t\right)\right)+\mathrm{sat}\left(\omega_{i1}\left(t\right)\right),0,0\right]}^{T}$, where $\mathrm{sat}\left(y\left(t\right)\right)=0.5\left(\left|y(t)+1\right|-\left|y(t)-1\right|\right)$. According to the control protocol designed in (4), Figure 3 describes an impulsive sequence with attack strength $\hat{d}=0.34$ and impulsive control gain $U_{k}=-0.35$. On the one hand, we assume that $\hat{\omega }=1.2$, $P=I_{12}$, $\xi_{i}\left(t\right)={\left[\xi_{i1}\left(t\right),\xi_{i2}\left(t\right),\xi_{i3}\left(t\right)\right]}^{T}$ and $\xi_{i}\left(t\right)={\left[0.15\cos\left(t\right),-0.2\sin\left(t\right),0.3\cos\left(t\right)\right]}^{T}$, then $\bar{\xi }=0.53$.

Considering the influence of an impulsive attack sequence, we adopt a distinctive impulsive signal ${\left\{t_{k}\right\}}_{k=1}^{+\infty }$, which satisfies (8) and is described as follows:

(40) $$\begin{array}{c} t_{k}-t_{k-1}=\left\{\begin{array}{c} \tilde{\chi },\;\;\;\;\;\;\;\;\mathrm{if}\;\mathrm{mod}\left(k,N_{0}\right)\neq 0,\\ N_{0}\left(\tau_{ave}-\tilde{\chi }\right)+\tilde{\chi },\;\;\mathrm{if}\;\mathrm{mod}\left(k,N_{0}\right)=0. \end{array}\right. \end{array}$$

where $\tilde{\chi }$ and $\tau_{ave}$ are positive numbers that satisfy $\tilde{\chi }\leq \tau_{ave}$, $N_{0}\in N$. Hence, we find $\tau_{\inf}=\inf_{k\in N}\left\{t_{k+1}-t_{k}\right\}=\tilde{\chi }$ and $\tau_{\sup}=\sup_{k\in N}\left\{t_{k+1}-t_{k}\right\}=N_{0}\left(\tau_{ave}-\tilde{\chi }\right)+\tilde{\chi }$. We choose $\tilde{\chi }=0.2,N_{0}=3,\tau_{ave}=0.4$, according to (40), and we find that $\tau_{\inf}=0.2$, $\tau_{\sup}=0.8$. Choose that $\kappa_{1}=\kappa_{2}=0.5,\varepsilon_{1}=\varepsilon_{2}=\varepsilon_{3}=1$, then κ=0.5. The parameters of system are set as follows:

$$A=\left[\begin{array}{ccc} -1.55 & 1.74 & 0\\ 1 & -1 & 1\\ 0.1 & -1.8 & 0.1 \end{array}\right],$$

with parameters T=diag{0.4,0.3,0.3}, Q=diag{0.6,0.2,0.5} and B(t)=diag{0.2cos(t),−1.5cos(t),0.4cos(t)}. In addition, let E[α(t)]=α=0.5, E[β(t)]=β=0.3 and $\bar{\psi }=0.5$.

Based on the designed parameters and in consideration of $\int_{s}^{t}H(v)dv\leq 0.1\left(t-s\right)+0.65$, then γ=0.1, $\gamma_{0}=0.65$. $\sigma =d^{2}\left(\varepsilon_{3}qb^{-1}+1\right)=0.2$, $\theta =\frac{\ln(Z_{\sup}\sigma )}{\tau_{\sup}}+\gamma =-1.037<0$.

**Figure 4:**
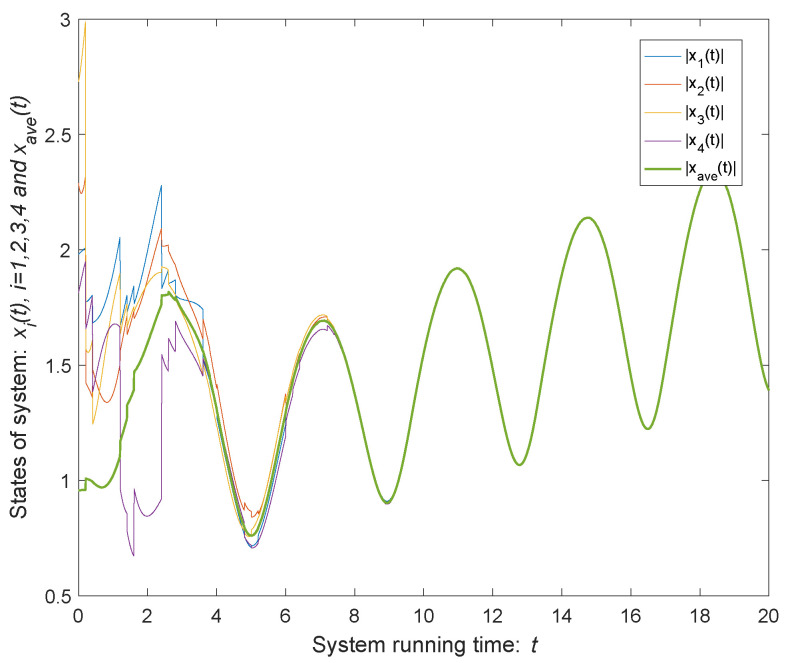
State of four agents.

**Figure 5:**
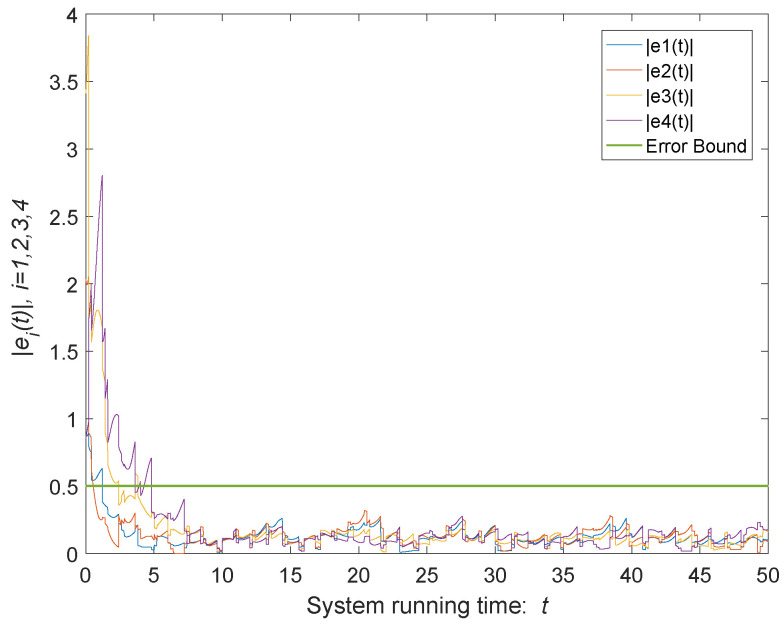
Trajectories of system errors under external inputs and impulse deception attacks.

**Figure 6:**
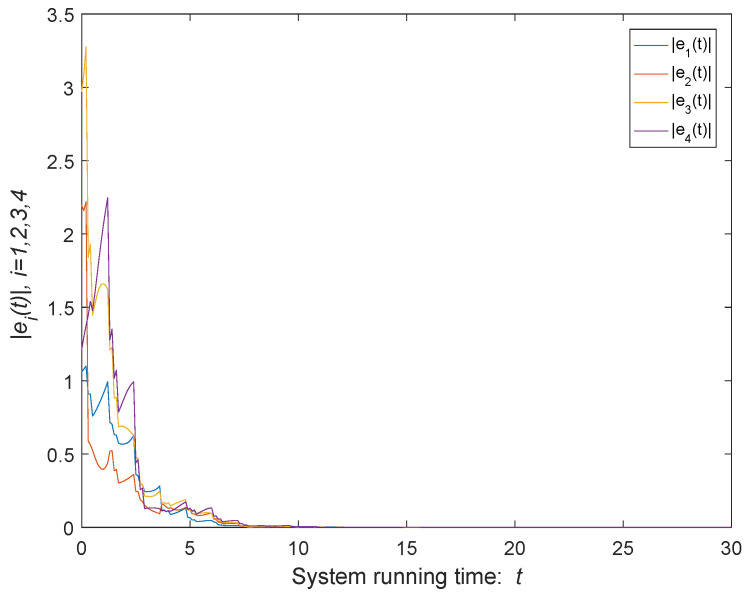
Trajectories of system errors without external inputs or impulse deception attacks.

As shown in Figure 4, the green curve represents the modulus of the average states of the agents of the time-varying MASs. According to the parameters selected above, the upper bound of error can be calculated ϑ=0.5, which is shown as Figure 5. When t→+∞, the trajectories of states coincide and the MASs achieves consensus. It can be seen from Figure 5 that, when system (1) disturbed by both external disturbances and impulse deception attacks, the quasi-consensus can be achieved under the control protocol (4), and the error is kept within the error bound. If there is no external disturbances or impulse deception attacks, the consensus of the system can be obtained as shown in Figure 6.

## 5. Conclusions

In this paper, we studied the quasi-consensus of a class of time-varying MASs suffering from both external inputs and deception attacks. By utilizing the analysis method from [5], we relaxed the restrictive assumption on time-varying matrices. To describe the success of deception attacks, a stochastic variable that obeys a Bernoulli distribution was adopted. By employing the comparison principle, sufficient conditions to ensure quasi-consensus were derived. Finally, a simulation example was given to verify the theoretical results.

## Data Availability

Not applicable.

## Abbreviations

The following abbreviations are used in this manuscript:

MASs	Multi-agent systems
DoS	Denial-of-service
